# Rapid, accurate, and novel diagnostic technique for respiratory pathogens: Clinical application of loop-mediated isothermal amplification assay in older patients with pneumonia, a multicenter prospective observational study

**DOI:** 10.3389/fmicb.2022.1048997

**Published:** 2022-12-19

**Authors:** Shanchen Wei, Lina Wang, Mingwei Shi, Jun Li, Chunping Sun, Yingying Liu, Zhi Zhang, Yiqun Wu, Lei Huang, Fei Tang, Liping Lv, Xiangdong Mu, Wei Tian, Caiwei Lin, Jianrong Lu, Baojun Sun, Bin Dai, Hui Xiong, Xiuhong Nie, Weimin Ding, Yuqing Ouyang, Lianjun Lin, Xinmin Liu

**Affiliations:** ^1^Department of Geriatrics, Peking University First Hospital, Beijing, China; ^2^Bio Biological Group Co., Ltd, Beijing, China; ^3^School of Public Health, Peking University Health Science Center, Beijing, China; ^4^Department of Clinical Laboratory, Peking University First Hospital, Beijing, China; ^5^Department of Respiratory, Anhui Chest Hospital, Hefei, China; ^6^Department of Respiratory, Tsinghua ChangGung Hospital, Beijing, China; ^7^Department of Geriatrics, Jishuitan Hospital, Beijing, China; ^8^Department of Emergency, Aerospace Center Hospital, Beijing, China; ^9^Department of Emergency, Jingmei Group General Hospital, Beijing, China; ^10^Department of Respiratory, Chinese People's Liberation Army General Hospital, Beijing, China; ^11^Department of Neurosurgery, Shijitan Hospital, Beijing, China; ^12^Department of Emergency, Peking University First Hospital, Beijing, China; ^13^Department of Respiratory, Xuanwu Hospital, Beijing, China; ^14^Department of Respiratory Endoscopy, Beijing Chest Hospital, Beijing, China

**Keywords:** loop-mediated isothermal amplification, chip, pneumonia, clinical, application, diagnostic, respiratory, pathogens

## Abstract

**Background:**

Loop-mediated isothermal amplification (LAMP) is a novel nucleic acid amplification method using only one type of enzyme that can amplify DNA with high specificity, efficiency and rapidity under isothermal conditions. Chips for Complicated Infection Detection (CCID) is based on LAMP. This study translate CCID into clinical application and evaluate its diagnostic value for pneumonia.

**Methods:**

Eighty one older patients with pneumonia were prospectively enrolled from January 1 to July 23, 2021, and 57 sputum/airway secretion and 35 bronchoalveolar lavage fluid samples were collected and analyzed by CCID and conventional microbiological tests (CMTs). Samples were collected, transported, monitored, and managed by a multidisciplinary team using a sample management information system.

**Results:**

CCID turnaround time was 50 min, and the detection limit was 500 copies/reaction. The percentage of positive samples was significantly higher using CCID than CMTs, especially for *Klebsiella pneumoniae* (odds ratio [OR], 9.0; 95% confidence interval [CI], 1.1–70.5; *p* < 0.05), *Enterococcus faecalis* (OR, ∞; *p* < 0.01), *Stenotrophomonas maltophilia* (OR, ∞; *p* < 0.01), fungi (OR, 26.0; 95% CI, 3.6–190.0; *p* < 0.01), and viruses (CCID only; *p* < 0.01). In addition, the percentage of positive results was significantly higher using CCID than CMTs in patients who used antibiotics for more than 3 days (91.9% vs. 64.9%; *p* < 0.01). Analyzing clinical impact, 55 cases (59.8%) benefited from CCID.

**Conclusion:**

CCID allows the rapid and accurate detection of pneumonia in older patients. Moreover, this technique is less affected by previous antibiotic treatment and can improve patient care.

## Introduction

The incidence of pneumonia is high in the elderly, which is a public health problem due to high mortality (Welte et al., 2012; Millett et al., 2013; Luna et al., 2016). Therefore, an accurate and rapid diagnosis is essential to identify the etiological agent, initiate targeted treatment, and reduce the mortality (Garau et al., 2008). However, conventional microbiological tests (CMTs) used in clinical practice have disadvantages. For instance, the detection rate of pathogens using CMTs is 27·4% (Lidman et al., 2002); among these, sputum culture has a long turnaround time (≥2 days), and the detection rate is 26–30% (Garcia-Vazquez et al., 2004; Signori et al., 2008; Shariatzadeh and Marrie, 2009). Therefore, there is an urgent need to find new diagnostic methods.

In 2019, the American Thoracic Society and the Infectious Diseases Society of America published guidelines and pointed out that rapid, cost-effective, sensitive, and specific diagnostic tests are key to supporting targeted therapies for community-acquired pneumonia and improving prognosis (Metlay et al., 2019). In recent years, the development and implementation of molecular diagnostic methods for pneumonia have improved clinical diagnosis. Nucleic acid amplification is one of the most valuable tools, and in addition to the widely used PCR-based assays (Saiki et al., 1985, 1988), several amplification methods have been invented, including nucleic acid sequence-based amplification (NASBA; Compton, 1991), self-sustained sequence replication (3SR) (Guatelli et al., 1990) and strand displacement amplification (SDA; Walker et al., 1992a,b). Each of these amplification methods has its own shortcomings when applied to the clinic. For example, the requirement of high-precision thermal cyclers in PCR prevents this powerful method from being widely used. NASBA and 3SR, which do not use thermal cycling, have weak points as well: increased backgrounds due to digestion of irrelevant DNA contained in the sample and the necessity to use costly modified nucleotides as substrate. Excitingly, a novel method called loop-mediated isothermal amplification (LAMP) is invented, which employs a type of DNA polymerase and a set of four specially designed primers (Notomi et al., 2000). In LAMP cycling, one inner primer hybridizes to the loop on the product and initiates displacement DNA synthesis, yielding the original stem–loop DNA and a new stem–loop DNA with a stem twice as long. Thus it can amplify DNA with high specificity, efficiency and rapidity under isothermal conditions (Notomi et al., 2000).

Chips for Complicated Infection Detection (CCID), developed by the Bio Biological Group Co., Ltd., uses LAMP-based microfluidic chip platforms (Huang et al., 2011). One such chip detecting eight respiratory bacteria has China Food and Drug Administration’s medical device registration (Registration No. 20173401346). Their previous data found that compared with DNA sequencing, the sensitivity and specificity of CCID are over 95 and 99%, respectively (Hou et al., 2018). However, the application value of CCID has not been verified by rigorous clinical studies. To achieve this goal, a prospective multicenter observational study was performed. CCID is transformed into clinical application and assessed the clinical value of diagnosis and treatment by a multidisciplinary team. We want to develop a rapid, accurate, and novel diagnostic technique for respiratory pathogens to improve the management of older patients with pneumonia.

## Materials and methods

### Participants and ethics

Patients diagnosed with pneumonia were prospectively recruited in 10 hospitals in China— Peking University First Hospital, Jishuitan Hospital, Xuanwu Hospital, Shijitan Hospital, Tsinghua ChangGung Hospital, Jingmei Group General Hospital, Aerospace Center Hospital, Beijing Chest Hospital, Chinese People’s Liberation Army General Hospital, and Anhui Chest Hospital from January 1, 2021, to July 23, 2021. According to the 2019 and 2007 Official Clinical Practice Guidelines of the American Thoracic Society and Infectious Diseases Society of America (Mandell et al., 2007; Metlay et al., 2019), pneumonia was defined as (1) cough, expectoration, or worsening symptoms of existing respiratory diseases with or without purulent sputum, chest pain, dyspnea, and hemoptysis; (2) fever; (3) signs of lung consolidation or rales; (4) peripheral blood leukocytes >10 × 10^9^ or < 4 × 10^9^ per liter with or without a shift to the left. The inclusion criteria were patients aged ≥60 years, at least one of the features above, and the presence of patchy infiltrates, consolidation, ground-glass opacities, or interstitial changes with or without pleural effusion on chest imaging. The exclusion criteria were pregnant women, patients unable to give written informed consent, patients unable to produce sputum spontaneously, and patients with tuberculosis, lung tumors, non-infectious interstitial lung diseases, pulmonary edema, atelectasis, pulmonary embolism, pulmonary eosinophil infiltration, or pulmonary vasculitis.

The research ethics committee of our institution approved the study protocol (Number 2021–132). Written informed consent was obtained from all participants.

### Data collection and case discussion

Demographic data, clinical manifestations, vital signs, laboratory indicators, CMT and imaging results, and antibiotic data were collected. Chinese infectious disease experts, laboratory personnel, clinicians, and researchers reached consensus on the etiological agent.

### Sample collection and CMTs.

Sputum and BALF samples were collected in accordance with clinical guidelines (Hospital infection control branch of Chinese preventive medicine association, 2018; Wang et al., 2019). The quality of sputum samples was verified by sputum smears. The results showed that the count of leukocytes was greater than 25 per low-power field, and the count of epithelial cells was less than 10 per low-power field, or the ratio of leukocytes to epithelial cells was greater than 2.5, indicating that the sputum samples were qualified. Bacterial pneumonia was confirmed by the presence of Gram-positive or Gram-negative bacteria on smears or routine cultures (at least10^4^ CFU/ml). Viral pathogens were screened by PCR. Fungi were detected by sputum smear microscopy, culture, or the detection of (1, 3)-β-D-glucan and galactomannan antigens. Pulmonary tuberculosis was confirmed by positive sputum smears for acid-fast bacilli or positive sputum culture for Mycobacterium tuberculosis.

CMTs were performed according to the Guidelines for the Diagnosis and Treatment of Chinese Adult Community-Acquired Pneumonia (2016 Edition) (Qu and Cao, 2016).

### Sample preparation and DNA extraction

Sputum and BALF samples were transferred to sterile test tubes and analyzed by CCID and CMTs. For CCID, the volume of sputum needed to be at least 2 ml, and the volume of BALF needs to be at least 3 ml. Samples were pretreated with an equal volume of 4% NaOH, vortexed for 15 min, and centrifuged at 12,000 rpm for 5 min. The supernatant was discarded, and Tris-EDTA buffer solution and glass beads were added to the pellet. The test tubes were centrifuged at 1,000 rpm for 5 min and heated at 95°C for 5 min to release nucleic acids. After centrifugation at 12,000rpm for 5 min, 25 μl of the supernatant was mixed with 25 μl of the amplification reagent, and the mixture was added to the chip. Assays were performed using the Pathogenic Bacteria Nucleic Acid Detection Kit (CapitalBio Technology, Beijing, China) following the manufacturer’s instructions.

### Pathogens detection

**Figure 1:**
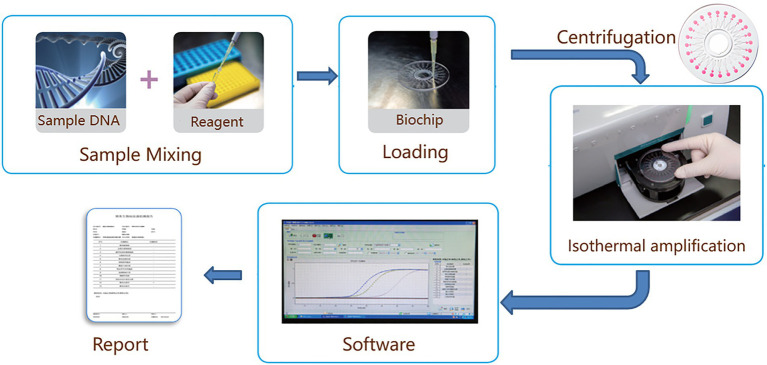
Experimental setup using the RTisochip amplification system.

**Figure 2:**
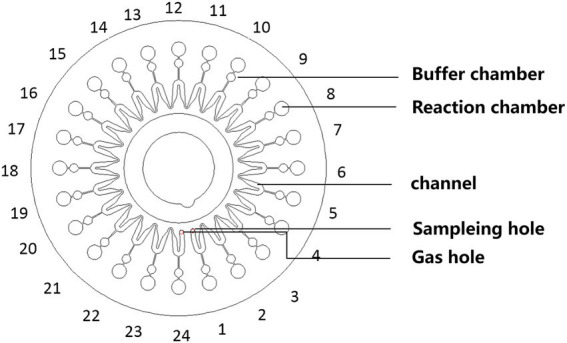
Schematic of the microfluidic chip consisting of an array of 24 reaction wells and microchannel for sample inlet, distribution, and reaction.

The schematic and amplification curves for pathogens detection were shown in Figure 1. The disc chip was used in this study, and its structure was shown in Figure 2. The process from sample loading to completion of amplification was shown in the animation (Supplementary material 1). The chip was centrifuged at 3,000 rpm for 30 s, making the mixture drop into the bottom of the reaction wells. Air trapped in the chip was released through air vents of each well, and inlet ports were covered to prevent contamination. Reactions were performed on an RTisochip-A nucleic acid analyzer equipped with a real-time imaging system (Figure 1; CapitalBio Technology, Beijing, China). The amplification conditions consisted of 1 cycle at 37°C for 3 min and 1 cycle at 65°C for 47 min. Data were analyzed using nucleic acid detection software. The CCID panel could detect 47 bacterial species, 22 fungal species, 21 DNA viruses, and 37 antibiotic resistance genes (Supplementary material 2).

### Determination of clinical impact

A positive impact was defined as a definitive diagnosis, supporting empirical antibiotic treatment, or change in therapeutic management based on CCID results, leading to a favorable clinical outcome. No impact was defined as valueless results or detecting no pathogens using CCID.

### Statistics analysis

Statistical analysis was performed using SPSS version 26.0 and R software version 3.6.1, and data were transferred to Excel spreadsheets. Data were expressed as mean ± standard deviation for normally distributed continuous variables and as median (interquartile range) for non-normally distributed continuous variables. Categorical data were presented as percentages. The difference in the detection rates between CCID and CMTs was analyzed by Pearson chi-square test, Fisher exact test, or McNemar test for discrete variables. A two-tailed *p* < 0.05 was considered statistically significant.

## Results

### Sample management information system and efficacy of CCID

Sample collection, transport, monitoring, and management were performed by a multidisciplinary team using a sample management information system.^1^. The turnaround time of CCID was 50 min, and the limit of detection was 500 copies per reaction.

### Baseline statistics

**Figure 3:**
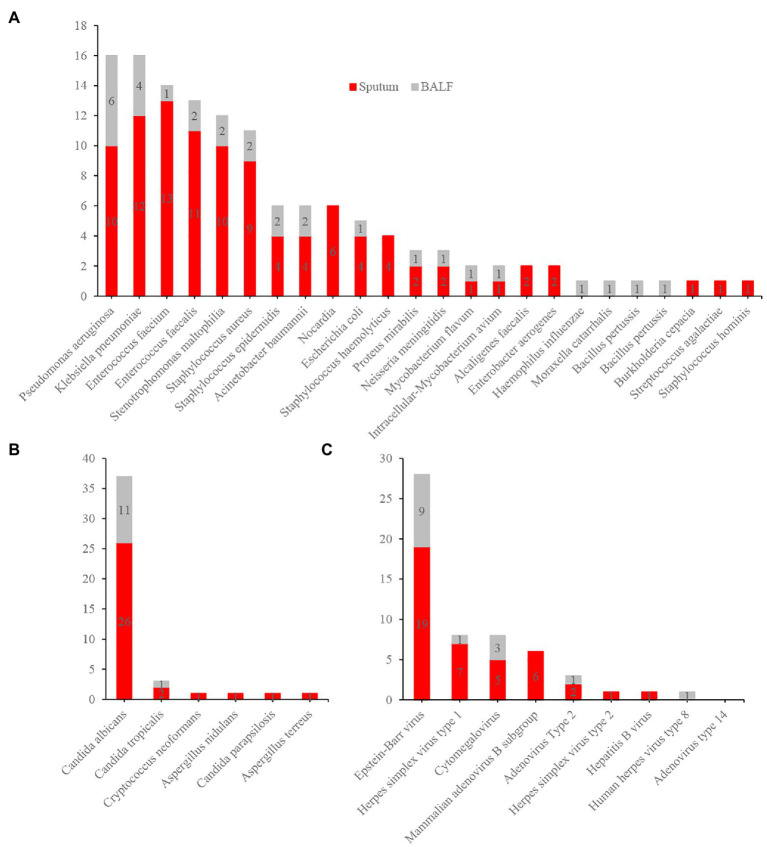
Distribution of bacteria **(A)**, fungi **(B)**, and virus **(C)** detected by Chips for Complicated Infection Detection. The most commonly detected bacteria, fungi, and virus are *Pseudomonas aeruginosa*, *Candida albicans*, and Epstein–Barr virus.

The demographic and clinical characteristics of our cohort are presented in Table 1. The median age was 78 (67.5–85.5) years. Fifty-eight patients were male, and 23 were female. The most common comorbidity was hypertension (43, 53.1%). The main clinical manifestations were cough (58, 71.6%), expectoration (55, 67.9%), fever (44, 54.3%), dyspnea (31, 38.3%), and disturbance of consciousness (18, 22.2%). Eighteen patients (22.2%) had respiratory failure, and 36 (44.4%) had severe pneumonia. As of July 23, 2021, 18 patients (22.2%) had died (Table 1). Ninety-two respiratory samples were collected from 81 patients. Sputum and bronchoalveolar lavage fluid (BALF) from the same person were submitted for examination in nine cases (9.8%), and two (2.2%) patients repeatedly submitted samples for examination during disease progression. Eighty-one samples (54 [66.7%] sputum and 27 [33.3%] BALF specimens) were analyzed by CCID and CMTs; of these, 44 (54.3%) and 37 (45.7%) were obtained from patients treated with antibiotics for ≤3 and > 3 days, respectively. The bacterial species most frequently detected by CCID were *Pseudomonas aeruginosa, Klebsiella pneumoniae, Enterococcus faecalis, Enterococcus faecium, Stenotrophomonas maltophilia*, and *Staphylococcus aureus* (Figure 3A). The most common fungal species was *Candida albicans* (Figure 3B), and the most common viruses were Epstein–Barr virus, herpes simplex virus type 1, cytomegalovirus, and mammalian adenovirus type B (Figure 3C).

**Table 1:** Demographic and clinical characteristics of the 81 patients included in the study at baseline.

**Characteristics**	**No. (%)**
Age, median (IQR), *y*	78 (67.5–85.5)
Sex	
Female	23 (28.4)
Male	58 (71.6)
History of underlying disease	
Hypertension	43(53.1)
Cardiac diseases	37 (45.7)
Cerebrovascular diseases	22 (27.2)
Diabetes	19 (235)
Neoplasia	10 (12.3)
COPD	8 (9.9)
Kidney disease	9 (11.1)
Autoimmune disease	6 (7.4)
Liver disease	2 (2 5)
Symptoms	
Cough	58 (71.6)
Expectoration	55 (67.9)
Fever	44 (54.3)
Dyspnea	31 (38.3)
Disorders of consciousness	18 (22.2)
Loss of appetite	16 (19.8)
Chest distress	15 (18.5)
Weak	9 (11.1)
Respiratory failure	18 (22.2)
Severe pneumonia	36 (44.4)
Outcome	
Dead	18 (22.2)
Survive	63 (77.8)

IQR, interquartile range; COPD, chronic obstructive pulmonary disease.

## Diagnostic performance

### Concordance between CCID and CMTs

**Figure 4:**
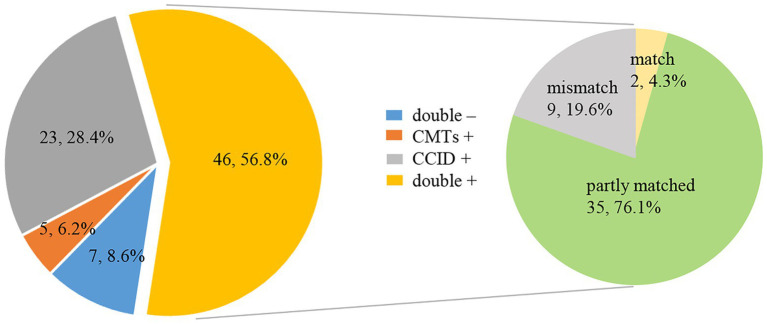
Pie chart demonstrating the positivity distribution and concordance between Chips for Complicated Infection Detection (CCID) and conventional microbiological tests (CMTs) for pneumonia in older people (*n* = 81). For the double-positive subset, a high proportion of partial matching (35/46) (at least 1 pathogen identified in the test was confirmed by the other) and complete matching (2/46) is seen, with only 9 conflicts between CCID and CMTs results.

Forty-six (56.8%) samples analyzed by CCID and CMTs were double positive, and seven (8·6%) were double negative. Twenty-three (28.4%) specimens were positive using CCID, and five (6·2%) were positive using CMTs. For double positive samples, there was complete concordance in two cases, discordance in nine cases, and partial concordance (diagnostic agreement for at least one pathogen) in 35 cases (Figure 4).

### Discordance between CCID and CMTs

Twenty-three (28.4%) samples were positive using CCID but negative using CMTs, of which more than half results (13/23) were regarded as pathogenic microbe by case discussion. The remaining cases were regarded as colonization, contamination, or other causes. In five (6.2%) specimens, pathogens were detected by CMTs but not by CCID (Table 2). Of these, one sample was positive by CCID in the second examination.

**Table 2:** Analysis of Inconsistent Results Between CCID and CMTs for Pathogen Detection.

***Pathogens Detected Only by CCID (N = 23)***					
**Sample No.**	**Sample types**	**Possible explanation**			
		**Pathogenic microbe**	**Likely colonization**	**Likely contamination**	**Other causes**
115	sputum	*Nocardia*			Epstein–Barr virus
117	sputum		Mammalian adenovirus B subgroup, herpes simplex virus type 1		*Nocardia*
118	sputum	*Candida albicans*, herpes simplex virus type 1, cytomegalovirus			mammalian adenovirus B subgroup
139	sputum		Epstein–Barr virus		
145	sputum	*Pseudomonas aeruginosa, Candida albicans*	*Stenotrophomonas maltophilia*	*Staphylococcus epidermidis*	
262	sputum			*Neisseria meningitidis*, Epstein–Barr virus, cytomegalovirus	*Enterobacter aerogenes*
295	sputum	*Staphylococcus aureus*		Epstein–Barr virus	*Candida albicans*
328	BALF		Epstein–Barr virus		
382	sputum			*Enterococcus faecalis*, *Staphylococcus epidermidis*, adenovirus type 2	
402	sputum			*Candida albicans*, Epstein–Barr virus	
447	sputum	*Pseudomonas aeruginosa*			
482	sputum			*Staphylococcus hominis*	Epstein–Barr virus
489	BALF	*Candida albicans*	Epstein–Barr virus		
529	BALF		*Candida albicans*		
582	sputum	*Candida albicans*			
594	sputum	*Klebsiella pneumoniae*			
683	sputum	*Acinetobacter baumannii*, *Candida albicans*	*Enterococcus faecalis*, *Enterococcus faecium*	Epstein–Barr virus	
691	BALF	*Acinetobacter baumannii*, *Candida tropicalis*	Epstein–Barr virus		*Enterococcus faecium*, *Candida albicans*
695	BALF		Epstein–Barr virus, herpes simplex virus type 1		
697	sputum	*Enterococcus faecium*, *Stenotrophomonas maltophilia*	herpes simplex virus type 1	*Staphylococcus epidermidis*	
807	BALF		*Bacillus pertussis*		
808	BALF	*Pseudomonas aeruginosa*, *Intracellular-Mycobacteria avium*			
836	sputum	*Escherichia coli*	*Cryptococcus neoformans*	Epstein–Barr virus	
***Pathogens detected only by CMTs (N = 5)***					
**Sample No.**	**Sample type**	**CMTs result**		**Possible explanation**	
321	sputum	Haemophilus parainfluenzae		Not detected	
366	BALF	*MTB*		Not detected	
377	BALF	*Staphylococcus aureus*, *MTB*		Not detected	
538	sputum	*Pseudomonas aeruginosa*		Repeat CCID test, *Pseudomonas aeruginosa* was detected.	
540	sputum	*Staphylococcus aureus*, *Klebsiella pneumoniae*		Not detected	

CCID, Chips for Complicated Infection Detection; CMTs, Conventional Microbiological Tests; BALF, Bronchoalveolar Lavage Fluid; MTB, Mycobacterium tuberculosis.

### Diagnostic comparison

**Figure 5:**
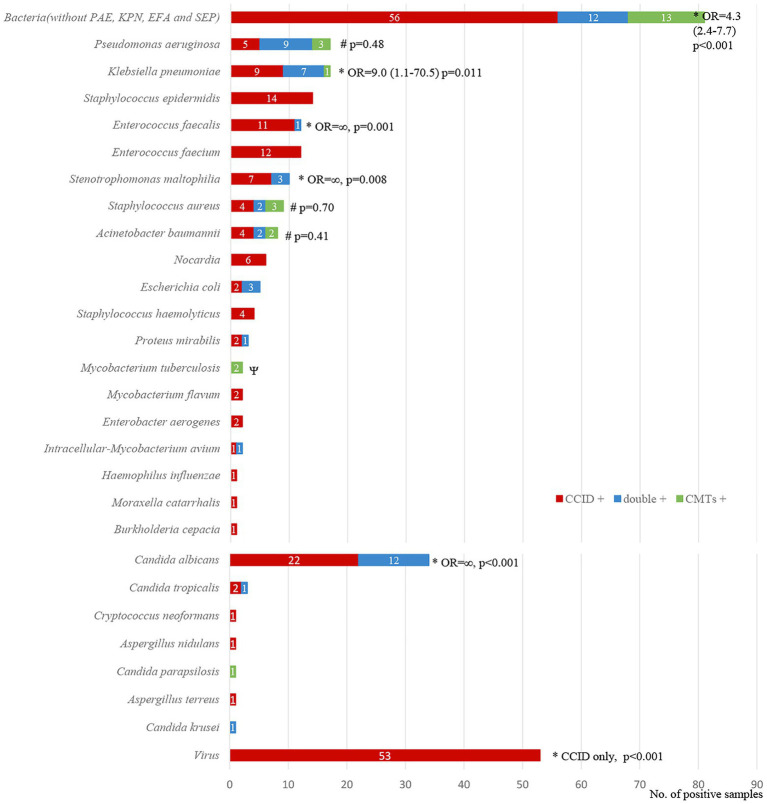
Diagnostic concordance between chips for complicated infection detection (CCID) and conventional microbiological tests (CMTs) for different pathogens. *^, #^The rates of positive results are significantly higher (*p* < 0.05) or marginally higher (*p* > 0.05) using CCID. OR, odds ratio.

Among 234 identified pathogens, CCID had higher ability to detect *Klebsiella pneumoniae* (odds ratio [OR], 9.0; 95% confidence interval [CI], 1.1–70.5; *p* = 0.011), *Enterococcus faecalis* (OR, ∞; *p* = 0.001), *Stenotrophomonas maltophilia* (OR, ∞; *p* = 0.008), fungi (OR, 26·0; 95% CI, 3·6–190.0; *p* < 0.001), and viruses (CCID only; *p* < 0.001). In addition, the rate of detection of *Pseudomonas aeruginosa*, *Staphylococcus aureus*, and *Acinetobacter baumannii* was marginally higher using CCID than CMTs (*p* > 0.05; Figure 5).

### Effect of antibiotic treatment on pathogen detection

**Figure 6:**
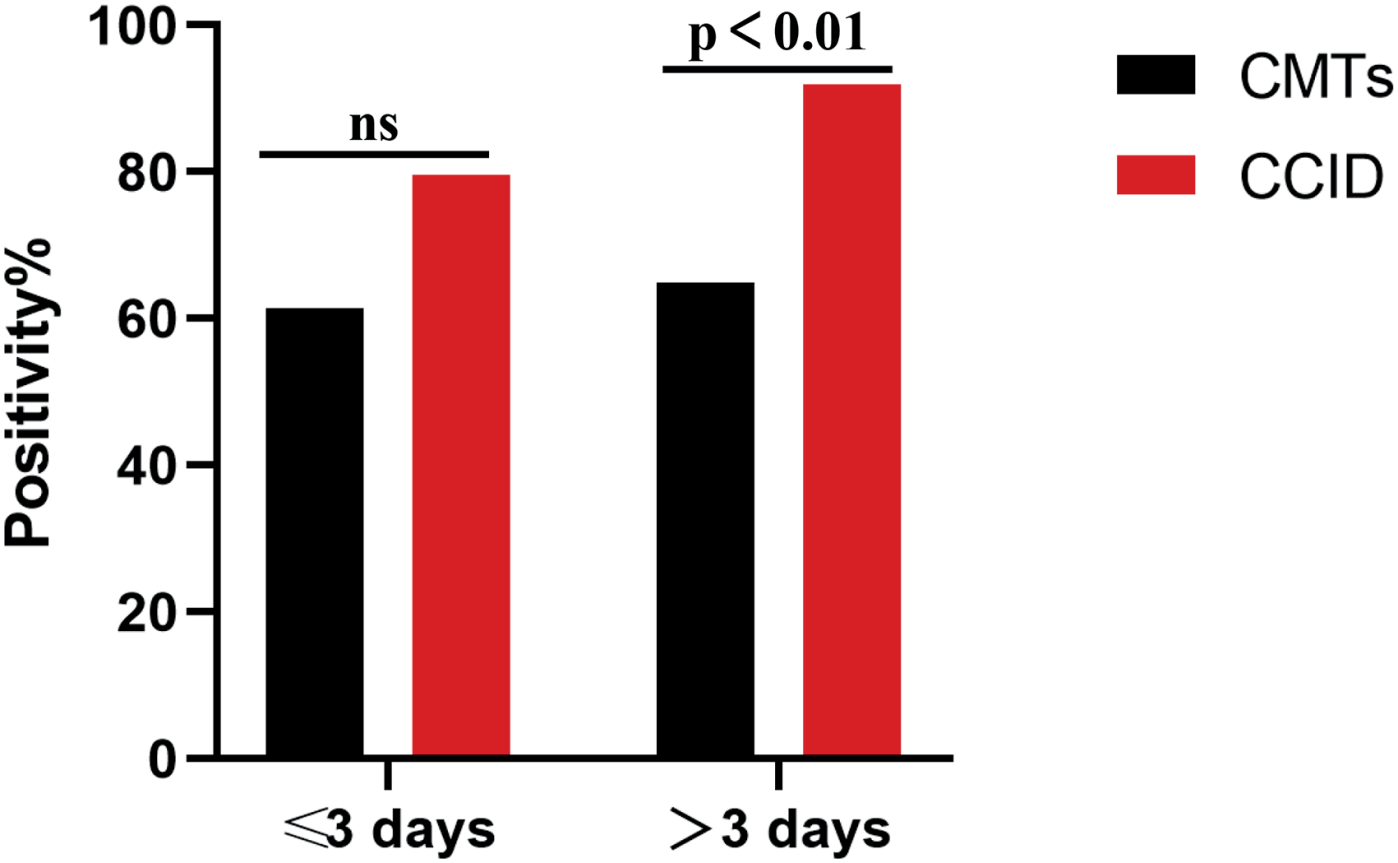
Percentage of positive samples in patients with pneumonia treated with antibiotics for ≤3 days and > 3 days. CCID, Chips for Complicated Infection Detection; CMTs, Conventional Microbiological Test; ns, no significance.

In our cohort, 37 (45.7%) patients used antibiotics for more than 3 days at the time of specimen collection. The detection rate was significantly higher using CCID than using CMTs (91.9% vs. 64·9%, *p* = 0.005). In the remaining patients, the detection rate was marginally higher using CCID (79.5% vs. 61.4%, *p* > 0.05) (Figure 6).

### Impact on clinical diagnosis and management

The analysis of the impact of CCID on patient care showed that this method had positive or no impact in 55 (59.8%) and 37 (40.2%) cases, respectively (Table 3). In the former group, positive CCID results allowed a definitive diagnosis in 55 patients. In the latter group, CCID did not detect the etiological agent in 16 patients, and the results were attributed to contaminants or other causes in 21 cases (Table 3).

**Table 3:** Clinical impact and role of CCID result.

**Clinical impact**	**Role of CCID result**	**Treatment changes due to CCID**
Positive impact (*n* = 55, 59.8%)	Contributed to definitive diagnosis (*n* = 55, 59.8%)	Empirical treatment continued (*n* = 51, 55.4%)
		Treatment adjusted (*n* = 4, 4.4%)
No impact (*n* = 37, 40.2%)	Results deemed valueless (*n* = 21, 22.8%)	No impact
	No pathogen detected (*n* = 16, 17.4%)	

CCID, Chips for Complicated Infection Detection; CMTs, Conventional Microbiological Tests.

Regarding antibiotic management in patients with a clinical benefit, CCID results supported empirical antibiotic treatment in 51 cases and led to a change in treatment in four cases (Table 3).

## Discussion

Loop-mediated isothermal amplification (LAMP) is a novel nucleic acid amplification method that can amplify DNA with high specificity, efficiency and rapidity under isothermal conditions (Notomi et al., 2000). Compared with several other methods, such as PCR, NASBA, 3SR and SDA, LAMP is more suitable for clinical application, since their instrumentation is simpler without high-precision thermal cyclers. Other key advantages of LAMP are robustness and the production of pyrophosphate in the presence of the target gene, enabling to detect the reaction products using the naked eye (Zhang et al., 2019). Polymerase inhibitors, presented in clinical samples, do not affect the amplification process, making LAMP suitable for a simple sample-to-answer diagnostic systems with simplified sample preparation (Zhang et al., 2019).

We searched PubMed database for articles with the keywords “LAMP, pneumonia or lower respiratory tract infections.” There was no language restriction, and COVID-19 was removed. We found three results (Hou et al., 2018; Scharmann et al., 2020; Wang et al., 2020), all three of which collected respiratory tract specimens and submitted them for LAMP and routine clinical pathogenic testing, but did not confirm and interpret the controversial results from a clinical perspective. The indicators detected by LAMP technology are limited to 2–9 kinds of bacteria, and no attention was paid to the elderly. While our study transformed CCID into clinical application. We found that CCID combined with LAMP has several advantages. First, CCID has a shorter turnaround time of 50 min than other diagnostic methods (Torres et al., 2016; Miao et al., 2018). Second, the percentage of positive samples was significantly higher using CCID than using CMTs. Among 81 samples, the results agreed between CCID and CMTs in 37 samples (two complete matches and 35 partial matches) and did not agree in 44 samples. In the latter cases, the causative agent was identified by consensus in 13 specimens and identified by CCID in one sample after repeating the test. Therefore, 51 (63·0%) samples analyzed by CCID had a clinical suggestion, and this rate is higher than that using CMTs (less than 36%; Haessler et al., 2020; Ogawa et al., 2020; Schimmel et al., 2020). Third, CCID had a higher ability to detect *Klebsiella pneumoniae, Enterococcus faecalis, Stenotrophomonas maltophilia*, fungi, and viruses. Fourth, CCID might have a wider scope of application because it was less affected by antibiotic treatment. Fifth, CCID had a clinical benefit in 59·8% of cases, including supporting empirical antibiotic treatment (55·4%) and adjustments in treatment (4·4%). Sixth, CCID is cheaper than other diagnostic methods (Supplementary material 3). A chip can cover 20 indicators and costs only 150–200 RMB.

However, the primers of CCID are specific for the target sequence, precluding the detection of rare subtypes. Moreover, high sensitivity allows identifying more than one pathogen, especially in sputum specimens, and diagnosis should be confirmed by computed tomography and clinical characteristics.

This prospective multicenter observational study standardized operations and procedures for sample collection and transportation and analyzed samples by CCID and CMTs in parallel to eliminate potential confounders. However, this study has limitations. First, the small sample size (92 samples from 10 hospitals) may lead to bias. Second, although standardized procedures were adopted, there is possibility of pollution in the actual operation process. Third, the viruses detected by CCID has not been confirmed. Fourth, CCID included 37 antibiotic resistance markers but disagreed with the results of CMTs.

Larger studies are necessary to resolve the discordant results by performing PCR, especially for the virus results. Regarding the detection of antibiotic resistance, although the discordance between genotype and phenotype is a shortcoming of molecular diagnostic technologies, genes associated with phenotypes can be identified using larger samples to improve clinical diagnosis. Microfluidic platforms can be customized for specific populations to detect a wide range of pathogen types, reduce unnecessary indicators, reduce costs, and improve the clinical utility of CCID. Moreover, pre-amplification for fungi and viruses should be performed to improve diagnostic sensitivity.

Our results showed that CCID was a promising method for accurate diagnosis and development of targeted antibiotic therapies.

## Data availability statement

The original contributions presented in the study are included in the article/Supplementary material, further inquiries can be directed to the corresponding authors.

## Ethics statement

The studies involving human participants were reviewed and approved by Ethics Committee of Peking University First Hospital. The patients/participants provided their written informed consent to participate in this study.

## Author contributions

XL and LL: conceived, designed, and supervised the study. SW, LW, MS, JL, and CS: acquired the data. SW, YW, LH, and YO: analyzed and interpreted the data. SW, FT, LL, XM, WT, CL, JL, BS, BD HX, XN, and WD: conducted the clinical work associated with the study. YL and ZZ: provided the technical support. SW, LL, and XL: verified the underlying data. SW: wrote the draft. LL: revised it. All authors read and approved the final version of the manuscript. The corresponding author attests that all listed authors meet the authorship criteria and that no others meeting the criteria have been omitted.

## Funding

This work was supported by National Key R&D Program of China [2020YFC2005401], Xicheng Financial, Scientific and Technological Project [XCSTS-SD2021-02], and Project funded by Baidu Fund of Peking University [2020BD045].

## Conflict of interest

YL was employed by CapitalBio Technology Co., Ltd. ZZ was employed by CapitalBio Corporation.

The remaining authors declare that the research was conducted in the absence of any commercial or financial relationships that could be construed as a potential conflict of interest.

## Publisher’s note

All claims expressed in this article are solely those of the authors and do not necessarily represent those of their affiliated organizations, or those of the publisher, the editors and the reviewers. Any product that may be evaluated in this article, or claim that may be made by its manufacturer, is not guaranteed or endorsed by the publisher.

## Footnotes

^1^https://smis.capitalbio.com and https://smis-m.capitalbio.com/

## References

[ref2] Compton J. (1991). Nucleic acid sequence-based amplification. Nature 350, 91–92. 1706072 10.1038/350091a0

[ref3] Garau J. Baquero F. Perez-Trallero E. Perez J. L. Martin-Sanchez A. M. Garcia-Rey C.. (2008). Factors impacting on length of stay and mortality of community-acquired pneumonia. Clin. Microbiol. Infect. 14, 322–329. doi: 10.1111/j.1469-0691.2007.01915.x 18190569

[ref4] Garcia-Vazquez E. Marcos M. A. Mensa J. de Roux A. Puig J. Font C.. (2004). Assessment of the usefulness of sputum culture for diagnosis of community-acquired pneumonia using the PORT predictive scoring system. Arch. Intern. Med. 164, 1807–1811. doi: 10.1001/archinte.164.16.1807, PMID: 15364677

[ref5] Guatelli J. C. Whitfield K. M. Kwoh D. Y. Barringer K. J. Richman D. D. Gingeras T. R. (1990). Isothermal, *in vitro* amplification of nucleic acids by a multienzyme reaction modeled after retroviral replication. Proc. Natl. Acad. Sci. U. S. A. 87, 1874–1878. doi: 10.1073/pnas.87.5.1874 2308948 PMC53586

[ref6] Haessler S. Lindenauer P. K. Zilberberg M. D. Imrey P. B. Yu P. C. Higgins T.. (2020). Blood cultures versus respiratory cultures: 2 different views of pneumonia. Clin. Infect. Dis. 71, 1604–1612. doi: 10.1093/cid/ciz1049 31665249 PMC7882190

[ref1] Hospital infection control branch of Chinese preventive medicine association (2018). Guidelines for collection and submission of clinical microbiological specimens]. Chin. J. Nosocomiol. 28, 3192–3200. doi: 10.11816/cn.ni.2018-183362

[ref7] Hou J. Wu H. Zeng X. Rao H. Zhao P. (2018). Clinical evaluation of the loop-mediated isothermal amplification assay for the detection of common lower respiratory pathogens in patients with respiratory symptoms. Medicine (Baltimore) 97:e13660. doi: 10.1097/MD.0000000000013660 30572483 PMC6320021

[ref8] Huang G. Wang C. Ma L. Yang X. Yang X. Wang G. (2011). Sensitive sequence-specific molecular identification system comprising an aluminum micro-nanofluidic chip and associated real-time confocal detector. Anal. Chim. Acta 695, 1–10. doi: 10.1016/j.aca.2011.03.040 21601025

[ref9] Lidman C. Burman L. G. Lagergren A. Ortqvist A. (2002). Limited value of routine microbiological diagnostics in patients hospitalized for community-acquired pneumonia. Scand. J. Infect. Dis. 34, 873–879. doi: 10.1080/0036554021000026967, PMID: 12587618

[ref10] Luna C. M. Palma I. Niederman M. S. Membriani E. Giovini V. Wiemken T. L.. (2016). The impact of age and comorbidities on the mortality of patients of different age groups admitted with community-acquired pneumonia. Ann. Am. Thorac. Soc. 13, 1519–1526. doi: 10.1513/AnnalsATS.201512-848OC, PMID: 27398827

[ref11] Mandell L. A. Wunderink R. G. Anzueto A. Bartlett J. G. Campbell G. D. Dean N. C.. (2007). Infectious Diseases Society of America/American Thoracic Society consensus guidelines on the management of community-acquired pneumonia in adults. Clin. Infect. Dis. 44, S27–S72. doi: 10.1086/511159 17278083 PMC7107997

[ref12] Metlay J. P. Waterer G. W. Long A. C. Anzueto A. Brozek J. Crothers K.. (2019). Diagnosis and treatment of adults with community-acquired pneumonia. An official clinical practice guideline of the American thoracic society and infectious diseases society of America. Am. J. Respir. Crit. Care Med. 200, e45–e67. doi: 10.1164/rccm.201908-1581ST 31573350 PMC6812437

[ref13] Miao Q. Ma Y. Wang Q. Pan J. Zhang Y. Jin W.. (2018). Microbiological diagnostic performance of metagenomic next-generation sequencing when applied to clinical practice. Clin. Infect. Dis. 67, S231–S240. doi: 10.1093/cid/ciy693 30423048

[ref14] Millett E. R. C. Quint J. K. Smeeth L. Daniel R. M. Thomas S. L. (2013). Incidence of community-acquired lower respiratory tract infections and pneumonia among older adults in the United Kingdom: a population-based study. PLoS One 8:e75131. doi: 10.1371/journal.pone.0075131, PMID: 24040394 PMC3770598

[ref15] Notomi T. Okayama H. Masubuchi H. Yonekawa T. Watanabe K. Amino N.. (2000). Loop-mediated isothermal amplification of DNA. Nucleic Acids Res. 28:E63. doi: 10.1093/nar/28.12.e63 10871386 PMC102748

[ref16] Ogawa H. Kitsios G. D. Iwata M. Terasawa T. (2020). Sputum gram stain for bacterial pathogen diagnosis in community-acquired pneumonia: a systematic review and Bayesian meta-analysis of diagnostic accuracy and yield. Clin. Infect. Dis. 71, 499–513. doi: 10.1093/cid/ciz876 31504334 PMC7384319

[ref17] Qu J. M. Cao B. (2016). Guidelines for the diagnosis and treatment of adult community acquired pneumonia in China (2016 edition). Zhonghua Jie He He Hu Xi Za Zhi 39, 241–242. doi: 10.3760/cma.j.issn.1001-0939.2016.04.005 27117069

[ref18] Saiki R. K. Gelfand D. H. Stoffel S. Scharf S. J. Higuchi R. Horn G. T.. (1988). Primer-directed enzymatic amplification of DNA with a thermostable DNA polymerase. Science 239, 487–491. doi: 10.1126/science.2448875 2448875

[ref19] Saiki R. K. Scharf S. Faloona F. Mullis K. B. Horn G. T. Erlich H. A.. (1985). Enzymatic amplification of beta-globin genomic sequences and restriction site analysis for diagnosis of sickle cell anemia. Science 230, 1350–1354. doi: 10.1126/science.2999980 2999980

[ref20] Scharmann U. Kirchhoff L. Schmidt D. Buer J. Steinmann J. Rath P. M. (2020). Evaluation of a commercial loop-mediated isothermal amplification (LAMP) assay for rapid detection of pneumocystis jirovecii. Mycoses 63, 1107–1114. doi: 10.1111/myc.13152 32738076

[ref21] Schimmel J. J. Haessler S. Imrey P. Lindenauer P. K. Richter S. S. Yu P. C.. (2020). Pneumococcal urinary antigen testing in United States hospitals: a missed opportunity for antimicrobial stewardship. Clin. Infect. Dis. 71, 1427–1434. doi: 10.1093/cid/ciz983 31587039 PMC7901240

[ref22] Shariatzadeh M. R. Marrie T. J. (2009). Does sputum culture affect the management and/or outcome of community-acquired pneumonia? East Mediterr. Health J. 15, 792–799. 20187530

[ref23] Signori L. G. Ferreira M. W. Vieira L. C. Muller K. R. Mattos W. L. (2008). Sputum examination in the clinical management of community-acquired pneumonia. J. Bras. Pneumol. 34, 152–158. doi: 10.1590/s1806-37132008000300005 18392463

[ref24] Torres A. Lee N. Cilloniz C. Vila J. Van der Eerden M. (2016). Laboratory diagnosis of pneumonia in the molecular age. Eur. Respir. J. 48, 1764–1778. doi: 10.1183/13993003.01144-2016 27811073

[ref25] Walker G. T. Fraiser M. S. Schram J. L. Little M. C. Nadeau J. G. Malinowski D. P. (1992a). Strand displacement amplification--an isothermal, in vitro DNA amplification technique. Nucleic Acids Res. 20, 1691–1696. doi: 10.1093/nar/20.7.1691 1579461 PMC312258

[ref26] Walker G. T. Little M. C. Nadeau J. G. Shank D. D. (1992b). Isothermal *in vitro* amplification of DNA by a restriction enzyme/DNA polymerase system. Proc. Natl. Acad. Sci. U. S. A. 89, 392–396. doi: 10.1073/pnas.89.1.392 1309614 PMC48243

[ref27] Wang G. F. Huang J. J. Zhang W. (2019). Guidelines for the application of diagnostic flexible bronchoscopy in adults (2019 edition). Zhonghua Jie He He Hu Xi Za Zhi 8, 573–590. doi: 10.3760/cma.j.issn.1001-0939.2019.08.005 31378019

[ref28] Wang Z. Zang Y. Gao Y. Han L. Lin H. Gao Y.. (2020). Evaluation of bronchoalveolar lavage fluid combined with the loop-mediated isothermal amplification assay in lower respiratory tract infections. Am. J. Transl. Res. 12, 4009–4016. 32774754 PMC7407691

[ref29] Welte T. Torres A. Nathwani D. (2012). Clinical and economic burden of community-acquired pneumonia among adults in Europe. Thorax 67, 71–79. doi: 10.1136/thx.2009.129502, PMID: 20729232

[ref30] Zhang H. Xu Y. Fohlerova Z. Chang H. Iliescu C. Neuzil P. (2019). LAMP-on-a-chip: revising microfluidic platforms for loop-mediated DNA amplification. Trends Analyt. Chem. 113, 44–53. doi: 10.1016/j.trac.2019.01.015 PMC7112807 32287531

