# Supplementary material for: Rapid, accurate, and novel diagnostic technique for respiratory pathogens: Clinical application of loop-mediated isothermal amplification assay in older patients with pneumonia, a multicenter prospective observational study
Source: Front Microbiol. 2022 Dec 19;13:1048997. doi: 10.3389/fmicb.2022.1048997 (PMC9806167; doi:10.3389/fmicb.2022.1048997)
Supplement: Supplementary file 1 [file Data_Sheet_1.PDF]

Bacteria targets			
**No.**	**Target**	**No.**	**Target**
1	*Staphylococcus aureus*	25	*Staphylococcus haemolyticus*
2	*Streptococcus pneumoniae*	26	*Staphylococcus sciuri*
3	*Klebsiella pneumoniae*	27	*Streptococcus agalactiae*
4	*Acinetobacter baumannii*	28	*Streptococcus dysgalactiae*
5	*Pseudomonas aeruginosa*	29	*Brucella*
6	*Haemophilus influenzae*	30	*Mycobacterium intracellulare/ avium*
7	*Stenotrophomonas maltophilia*	31	*Mycobacterium kanasii*
8	*Mycobacterium tuberculosis*	32	*Mycobacterium gordonae*
9	*Legionella pneumophila*	33	*Mycobacterium scrofulaceum*
10	*Escherichia coli*	34	*Mycolicibacterium phlei*
11	*Enterococcus faecalis*	35	*Mycobacterium gilvum*
12	*Enterococcus faecium*	36	*Mycobacterium chelonae/ abscessus*
13	*Burkholderia cepacia/Burkholderia cenocepacia*	37	*Mycobacterium marinum/ ulcerans*
14	*Alcaligenes faecalis*	38	*Serratia marcescens*
15	*Streptococcus pyogenes*	39	*Morganella morganii*
16	*Klebsiella oxytoca*	40	*Moraxella catarrhalis*
17	*Proteus mirabilis*	41	*Nocardia*
18	*Klebsiella aerogenes*	42	*Bordetella pertussis*
19	*Enterobacter cloacae*	43	*Chlamydia pneumoniae*
20	*Neisseria meningitidis*	44	*Mycoplasma pneumoniae*
21	*Clostridium perfringens*	45	*Treponema pallidum*
22	*Staphylococcus epidermidis*	46	*Borrelia burgdorferi*
23	*Staphylococcus capitis*	47	*Leptospira interrogans*
24	*Staphylococcus hominis*		

Fungi targets			
**No.**	**Target**	**No.**	**Target**
1	*Candida albicans*	12	*Cryptococcus gattii*
2	*Candida tropicalis*	13	*Rhizopus oryzae*
3	*Candida parapsilosis*	14	*Lichtheimia corymbifera*
4	*Candida kruseii*	15	*Rhizomucor pussilus*
5	*Candida glabrata*	16	*Aspergillus calidoustus*
6	*Aspergillus fumigatus*	17	*Pneumocystis jirovecii*
7	*Aspergillus flavus*	18	*Mucor circinelloides*
8	*Aspergillus niger*	19	*Histoplasma capsulatum*
9	*Aspergillus terreus*	20	*Penicillium marneffei*
10	*Aspergillus nidulans*	21	*Trichosporon asahii*
11	*Cryptococcus neoformans*	22	*Candida auris*

**Virus targets**			
**No.**	**Target**	**No.**	**Target**
1	*Human_mastadenovirus_B*	12	*Human gammaherpesvirus 8*
2	*Mastadenovirus type 2*	13	*Human papillomavirus 16*
3	*Mastadenovirus type 31*	14	*hepatitis B virus*
4	*Mastadenovirus type 40*	15	*simian virus 40*
5	*Mastadenovirus type 41*	16	*Mastadenovirus type 3*
6	*Mastadenovirus type 7*	17	*Mastadenovirus type 5*
7	*Human gammaherpesvirus 4/ Epstein-Barr virus*	18	*Mastadenovirus type 14*
8	*Human_alphaherpesvirus_1/ Herpes simplex virus 1*	19	*Mastadenovirus type 21*
9	*Human_alphaherpesvirus_2/ Herpes simplex virus 2*	20	*Mastadenovirus type 55*
10	*Human herpesvirus 5/ Human cytomegalovirus*	21	*Human bocavirus*
11	*Human herpesvirus 3/ Varicella zoster virus*		
**Resistance genes targets**			
**No.**	**Target**	**20**	**Target**
1	*aacC1*	21	*mefA*
2	*ermB*	22	*DHA-1*
3	*mecA*	23	*CMY-2*
4	*VanA*	24	*ACT*
5	*VanB*	25	*cfxa*
6	*OXA24*	26	*GES-1*
7	*OXA48/181*	27	*CTX-M1*
8	*OXA23*	28	*CTX-M9*
9	*OXA58*	*29*	*OXA-1*
10	*OXA66*	30	*OXA-10*
11	*IMP*	31	*tetC*
12	*VIM*	32	*tetW*
13	*KPC*	33	*tetQ*
14	*NDM*	34	*qnrA*
15	*SIM*	*35*	*qnrS*
16	*OprD*	*36*	*arr-2*
17	*TEM*	*36*	*mcr-1*
18	*SHV*	*37*	*aadA1*
19	*ompK35*		
