# Supplementary material for: Rapid, accurate, and novel diagnostic technique for respiratory pathogens: Clinical application of loop-mediated isothermal amplification assay in older patients with pneumonia, a multicenter prospective observational study
Source: Front Microbiol. 2022 Dec 19;13:1048997. doi: 10.3389/fmicb.2022.1048997 (PMC9806167; doi:10.3389/fmicb.2022.1048997)
Supplement: Supplementary file 2 [file Data_Sheet_2.PDF]

Supplemental Material 3

Cost of Each Method	
**Methods**	**Cost/RMB**
sputum smear	10
bacterial culture + identification	70 per bacterium
antimicrobial susceptibility tests	30 per tablet
acid-fast staining	20
fungal culture	70 per fungus
tuberculosis liquid culture	150
CCID	150-200 per chip (20 indicators)
mNGS	3000-4000

The cost mainly refers to the standard charges of the centers included in this study. CCID: Chips for Complicated Infection Detection; mNGS: metagenomic next-generation sequencing.
