# Supplementary material for: Rapid, accurate, and novel diagnostic technique for respiratory pathogens: Clinical application of loop-mediated isothermal amplification assay in older patients with pneumonia, a multicenter prospective observational study
Source: Front Microbiol. 2022 Dec 19;13:1048997. doi: 10.3389/fmicb.2022.1048997 (PMC9806167; doi:10.3389/fmicb.2022.1048997)
Supplement: Supplementary file 3 [file Presentation_1.PPTX]

## Slide 1
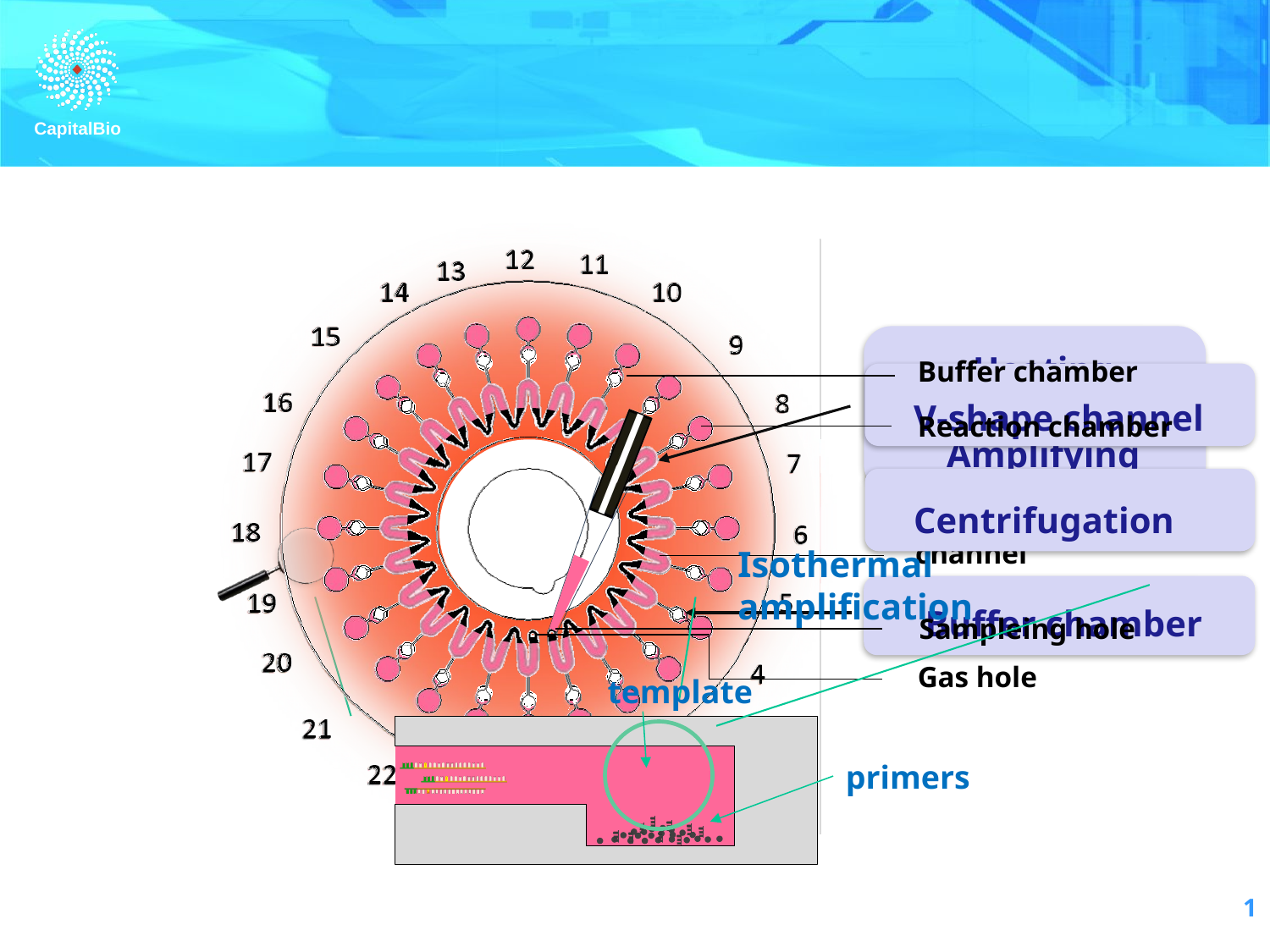

Time ≤ 50min
Heating
And
Amplifying
Buffer chamber
Reaction chamber
channel
Sampleing hole
Gas hole
V-shape channel
Centrifugation
Isothermal amplification
Buffer chamber
template
primers
1
